# Pediatric Vaccines and Neurodevelopment: Primate Study Finds No Adverse Behavioral Effects

**DOI:** 10.1289/ehp.123-A156

**Published:** 2015-06-01

**Authors:** Julia R. Barrett

**Affiliations:** Julia R. Barrett, MS, ELS, a Madison, WI–based science writer and editor, is a member of the National Association of Science Writers and the Board of Editors in the Life Sciences.

Vaccination has successfully reduced the incidence and prevalence of many infectious diseases. However, in the absence of outbreaks, a perception has developed that vaccinations themselves may present a greater risk than the diseases they prevent.^1^ Although evaluations of the existing data have failed to identify links between vaccination and negative health outcomes,^2^^,^^3^ there remain some public concerns about a potential link with autism spectrum disorders. These concerns are primarily due to the expansion of the infant immunization schedule and exposure to thimerosal, once used as a preservative in some vaccines.^1^ In this issue of *EHP*, the authors of a detailed exploration of neurodevelopment, learning, and social behavior in macaques report no adverse developmental and behavioral effects associated with the full pediatric vaccine schedule.^4^

Thimerosal is broken down in the body to thiosalicylate and ethylmercury.^5^ The latter is related to neurotoxic methylmercury, and estimates of health risks were based on the assumption—later disproved^6^—that ethyl- and methylmercury have similar toxicity profiles.^5^ With the exception of inactivated influenza vaccine, thimerosal has been removed from or reduced to trace amounts (≤ 1 µg/dose) in all vaccines routinely recommended for children under 6 years of age.^7^ Nevertheless, concerns persist, and vaccination rates have declined in some populations, with subsequent reemergence of diseases such as the recent outbreak of measles originating in California.^8^

**Figure:**
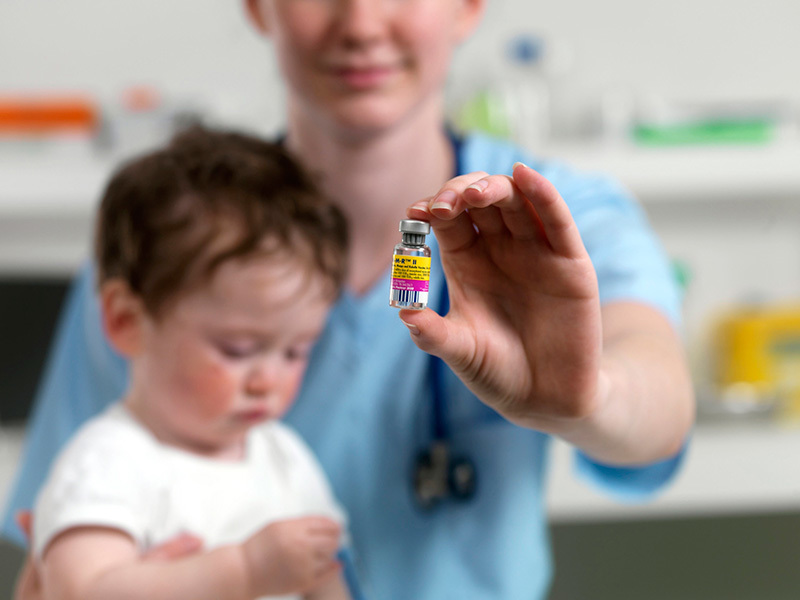
Results from a study of macaque neurodevelopment bolster the body of evidence that routine pediatric vaccines are safe for human children. © TEK IMAGE

“The main impetus behind the study was that while individual pediatric vaccines underwent the required clinical safety testing, the cumulative exposure to multiple thimerosal-containing vaccines had not been examined,” says study coauthor Laura Hewitson, director of research at the Johnson Center for Child Health and Development in Austin, Texas. The current study scrutinized neurodevelopment in macaques, whose nervous system development follows a similar trajectory to humans. Macaques also have learning and memory processes and social interactions that mimic those observed in humans.

Seventy-nine macaques were assigned to six vaccination groups: 1) controls—animals received saline injections only; 2) MMR—animals received only the measles, mumps, and rubella vaccine, which has never contained thimerosal; 3) TCV—animals received all the thimerosal-containing vaccines administered routinely to children in the 1990s^9^ but no MMR vaccine; 4) 1990s Pediatric—animals received all pediatric vaccines administered in the 1990s, including TCVs and MMR, on the schedule recommended for children; 5) 1990s Primate—animals received all pediatric vaccines administered in the 1990s on an accelerated schedule to match macaque development; and 6) 2008—animals received the expanded immunization schedule in place as of 2008,^10^ which is similar to the current schedule.

Between birth and 12 months of age, animals were tested for neonatal reflexes, object permanence (a measure of early memory development), discrimination learning strategies (ability to respond differently to different stimuli), and social behaviors. Animals also were systematically observed and rated for their level and characteristics of exploration, social withdrawal, play behavior, and aggression.

The investigators found that animals within each dosing group showed similar development of neonatal reflexes and object permanence. There was a slight indication that the TCV and 1990s Primate groups performed better in some learning tests, and some of the experimental groups demonstrated fewer negative social behaviors than the control group. However negative behaviors were rarely observed in any group, and learning and social development overall appeared normal.^4^

“Some analyses suggested a beneficial effect of the thimerosal exposure,” says Neal Halsey, director of the Institute for Vaccine Safety at the Johns Hopkins Bloomberg School of Public Health. However, says Halsey (who was not involved in the study), there currently is no plausible biologic mechanism to explain those associations, which makes it more likely they were found by chance.

“Most importantly, in terms of the behavioral data, we saw virtually no negative behaviors across all study groups,” says Hewitson. “That speaks to the hypothesis of thimerosal directly affecting behavior,” she says. “The fact that we didn’t see an increase in negative behaviors is reassuring.”
